# Non-invasive bladder volume measurement for the prevention of postoperative urinary retention: validation of two ultrasound devices in a clinical setting

**DOI:** 10.1007/s10877-018-0123-6

**Published:** 2018-03-07

**Authors:** Tammo A. Brouwer, Charina van den Boogaard, Eric N. van Roon, Cor J. Kalkman, Nic Veeger

**Affiliations:** 1 Department of Anaesthesiology, Medical Center Leeuwarden, Henri Dunantweg 2, PO Box 888, 8901 BR Leeuwarden, The Netherlands; 2 Institute for Science, Medical Center Leeuwarden, Henri Dunantweg 2, PO Box 888, 8901 BR Leeuwarden, The Netherlands; 3 Department of Pharmacotherapy, Epidemiology and Economics, University of Groningen, Groningen, The Netherlands; 4 Department of Clinical Pharmacy and Pharmacology, Medical Center Leeuwarden, Leeuwarden, The Netherlands; 5 Division of Anesthesiology, Intensive Care and Emergency Medicine, University Medical Center Utrecht, Utrecht, The Netherlands; 6 Clinical Epidemiologist, Department of Epidemiology, Medical Center Leeuwarden, Henri Dunantweg 2, PO Box 888, 8901 BR Leeuwarden, The Netherlands; 7 Department of Epidemiology, University Medical Center, Groningen, The Netherlands

**Keywords:** Bladder catheterization, Bladder volume, Post operative urinary retention, Ultrasound BladderScan, Validation

## Abstract

Ultrasound scanning of bladder volume is used for prevention of postoperative urinary retention (POUR). Accurate assessment of bladder volume is needed to allow clinical decision-making regarding the need for postoperative catheterization. Two commonly used ultrasound devices, the BladderScan® BVI 9400 and the newly released Prime® (Verathon Medical®, Bothell, WA, USA), with or without the ‘pre-scan’ option, have not been validated in clinical practice. The aim of this study was to assess the performance of these devices in daily clinical practice. Between June and September 2016 a prospective observational study was conducted in 318 surgical patients (18 years or older) who needed a urinary catheter perioperatively for clinical reasons. For acceptable performance, we required that the volume as estimated by the BladderScan® differs by no more than 5% from the actual urine volume after catheterization. The Schuirmann’s two one-sided test was performed to assess equivalence between the BladderScan® estimate and catheterization. The BVI 9400® overestimated the actual bladder volume by + 17.5% (95% CI + 8.8 to + 26.3%). The Prime® without pre-scan underestimated by − 4.1% (95% CI − 8.8 to + 0.5%) and the Prime® with pre-scan underestimated by − 6.3% (95% CI − 11.6 to − 1.1%). This study shows that while both ultrasound devices were able to approximate current bladder volume, both BVI 9400® and Prime®—with and without pre-scan—were not able to measure the actual bladder volume within our predefined limit of ± 5%. Using the pre-scan feature of the Prime® did not further improve accuracy.

## Introduction

Transabdominal ultrasound is frequently used for measuring bladder volumes non-invasively to prevent postoperative urinary retention (= POUR) by timely catheterization, but also to avoid unnecessary bladder catheterizations [1–4]. Bladder catheterization is the “standard” treatment for POUR [1, 5, 6], but it is an invasive procedure that contributes to an increased risk of urinary tract infections, urethral trauma, patient discomfort and unplanned and prolonged hospital admissions [7–10]. Adequate monitoring of postoperative bladder volumes is mandatory to limit the risks associated with prolonged bladder overdistension or bladder catheterization [1, 2, 11, 12].

The BladderScan® (Verathon**®**, Bothell, WA, USA) is a dedicated ultrasound device to clinically determine bladder volumes. The two latest editions of the BladderScan® are the BVI 9400® and its successor the Prime®. Although both devices are CE marked and widely used in hospitals, the performance of their algorithms has never been validated in a perioperative clinical setting. The Prime® has the possibility to use a pre-scan function, showing real time echo images of the bladder next to the normally displayed scanned bladder. The aim of this pre-scan function is to improve accuracy.

The manufacturer of the BladderScan® claims an accuracy of ± 15% ± 15 mL. We, a priori, defined adequate clinical performance when the estimated volume differs from the actual urine volume after catheterization by no more than 5%, based on clinical literature [13–15]. The aim of this study is to validate the performance of the BVI 9400® and the Prime® (*with* and *without* pre-scan) in surgical patients by assessing the difference between the BladderScan® estimate and the actual urine volume assessed by catheterization.

## Methods

### Design

After institutional ethical approval, an investigator initiated prospective study was conducted between June and September 2016 in three groups of at least 100 consecutive surgical patients, each requiring bladder catheterization. All patients gave written informed consent. Bladder volumes were assessed using either the non-invasive BladderScan BVI 9400® or the Prime®, *with* or *without* a pre-scan. Following the non-invasive estimation of the bladder volume all patients underwent urinary catheterization during which the actual urine volume was assessed as the “gold standard”.

### Patient selection procedure

Two groups of surgical patients (18 years or older, ASA classification I–IV), who required a perioperative bladder catheter following standard clinical hospital protocols, were included. The first group of patients consisted of surgical patients who needed a catheter after induction of anaesthesia and prior to the start of surgery due to expected volume shifts, epidural anaesthesia, or surgery in the smaller pelvis for example during coronary arterial bypass graft (CABG) surgery, orthopaedic hip or knee prostheses surgery or colonic surgery (“Perioperative Urinary Catheterization Protocol” Medical Center Leeuwarden). The second group of patients consisted of *post*operative patients at the post anaesthesia care unit (PACU) who were unable to void spontaneously and needed a catheter to prevent bladder overdistension (bladder volume ≥ 500 mL, “Micturition Protocol” Medical Center Leeuwarden). Exclusion criteria were a surgical incision in the suprapubic region, abdominal ascites, and pregnancy. Furthermore, surgical patients with an actual catheterized volume ≤ 30 mL in the operating theatre were excluded for analysis, as the BladderScan® is not sensitive to detect bladder volumes ≤ 30 mL (based on the information from the manufacturer).

### Sample size

Based on previous studies we hypothesized that the estimated volume by the BladderScan® devices differs from the volume after catheterization by no more than 5% [13, 15]. With the predefined margin of equivalence of ± 5% and an assumed correlation of 0.90, a sample size of at least 92 pairs per study group was needed to achieve 90% power to detect equivalence [16].

### Study procedure

The researchers (CB or TAB) or operation nurses in the operating theatre and PACU performed all the measurements. They were trained in the use of the Prime® by an instructor of the manufacturer. Included patients with a clinical indication for *intra*operative bladder catheterization were asked not to void shortly before the time of the surgery to ensure a measurable bladder volume. After induction of anaesthesia, three consecutive ultrasound measurements (scans) were performed in the supine position using the BladderScan® device in accordance with the cohort the patient was included. After three non-invasive measurements with each type of the BladderScan®, the patient was directly catheterized to measure the actual bladder volume. The first device to start with was the BVI9400®, by measuring bladder volumes non-invasively in 100 or more consecutive patients. After at least 100 patients were included with the BVI9400®, the next device used was the Prime® *without* pre-scan for measuring bladder volume in at least 100 consecutive patients. Again, after more than 100 patients were included with the Prime® *without* pre-scan, the Prime® *with* pre-scan function was used for measuring bladder volume non-invasively in at least 100 consecutive patients.

In included patients requiring *postoperative* bladder catheterization, the BVI 9400® was used at the PACU in accordance with the local hospital protocol (= *postoperative bladder catheterization is indicated when the scanned bladder volume is larger than the threshold of 500 mL and the patient is unable to void spontaneously*). If this was the case during the study period, then three consecutive measurements in the supine position were performed with the type of BladderScan® which was momentarily used in the *intra*operative group of surgical patents measured in the OR, which were in following order; the BVI 9400®, the Prime® *without* pre-scan and the Prime® *with* pre-scan.

With the BVI 9400® and Prime® *without* pre-scan, the aiming circle on the display was used to guide the position of the probe. In the Prime® *with* pre-scan, the “real echo” pre-scan image was developed to guide the position of the probe. By changing the position of the probe, a green line is trying to encircle the whole bladder echo image. If this was the case, this image could be fixed and a BladderScan was followed, assuming that the best image would lead to the best result = measuring the total bladder with the largest bladder volume. A print of each measurement was collected to assess which of the three measurements was the “best” BladderScan® estimate, defined as the largest and most centrally displayed bladder together with the highest scanned volume. In all patients, catheterization took place immediately after the scanning procedure. The actual catheterized urine volumes were collected in a calibrated bowl. A 60 mL syringe was used to retrieve the last millilitres of urine to assess the precise volume and to be sure the bladder was completely emptied.

### Outcomes

The primary outcome was the difference in volume in mL between the “best” estimated bladder volume measured with the BladderScan®, using the three different methods (BVI 9400®, Prime® *without* pre-scan, and Prime® *with* pre-scan) versus the actual measured urine volume after catheterization. In this, the difference between estimated and actual catheterized volume was expressed as the percentage of the actual catheterized volume.

Secondary outcomes included the difference in volume between the best estimated and the actual measured urine volume after catheterization over the small to large volume range (≤ 400 and > 400 mL). We choose 400 mL, because volumes smaller than 400 mL are considered safe, not leading to bladder distention. Above 400 mL, bladder volumes have to be measured non-invasively at regularly intervals to prevent bladder distention. In literature different volume limits are used for urinary retention (400, 500 or 600 mL) [3, 17, 18]. These commonly used volume limits are used to calculate the proportion of correct decisions to catheterize, after measuring the bladder volume non-invasively.

Furthermore, homogeneity of outcome in different patient subgroups was evaluated for gender, age, and BMI.

### Statistical analysis

All data are expressed as mean ± SD for normally distributed data and median with range for skewed data. Normality was assessed by visual inspection and the Shapiro–Wilk test.

For the primary outcome, the Schuirmann’s two one-sided test was used. For the secondary outcome, Bland–Altman analyses were performed to assess the level of agreement between the BladderScan® estimate and the actual volume after catheterization over the full range of volumes and over the two volume ranges of ≤ 400 and > 400 mL [19].

To evaluate the performance of the devices with regard to correct clinical decision making the proportion true positives and true negatives were analysed. A Student’s t test was used to evaluate differences between groups for normally distributed data, or a Mann–Whitney U test for non-normality. A one-way independent ANOVA or Kruskall-Wallis test was performed in case of a three-group comparison, depending on the normality of the data. For qualitative parameters, comparisons between groups were evaluated using Fisher exact test (dichotomous response) or Chi square test (exact when indicated). Preplanned subgroup analyses consisted of demographic characteristics that might influence the accuracy of the BladderScan®: gender (male vs. female), age (< 60 years vs. ≥ 60 years), and BMI (< 25 kg/m^2^ vs. 25–30 kg/m^2^ vs. ≥30 kg/m^2^). A 2-tailed p-value less than 0.05 was considered statistically significant. All analyses were performed using commercially available computer software (Statistical Analysis System version 9.4, SAS Institute, Cary, NC, USA).

## Results

**Fig. 1:**
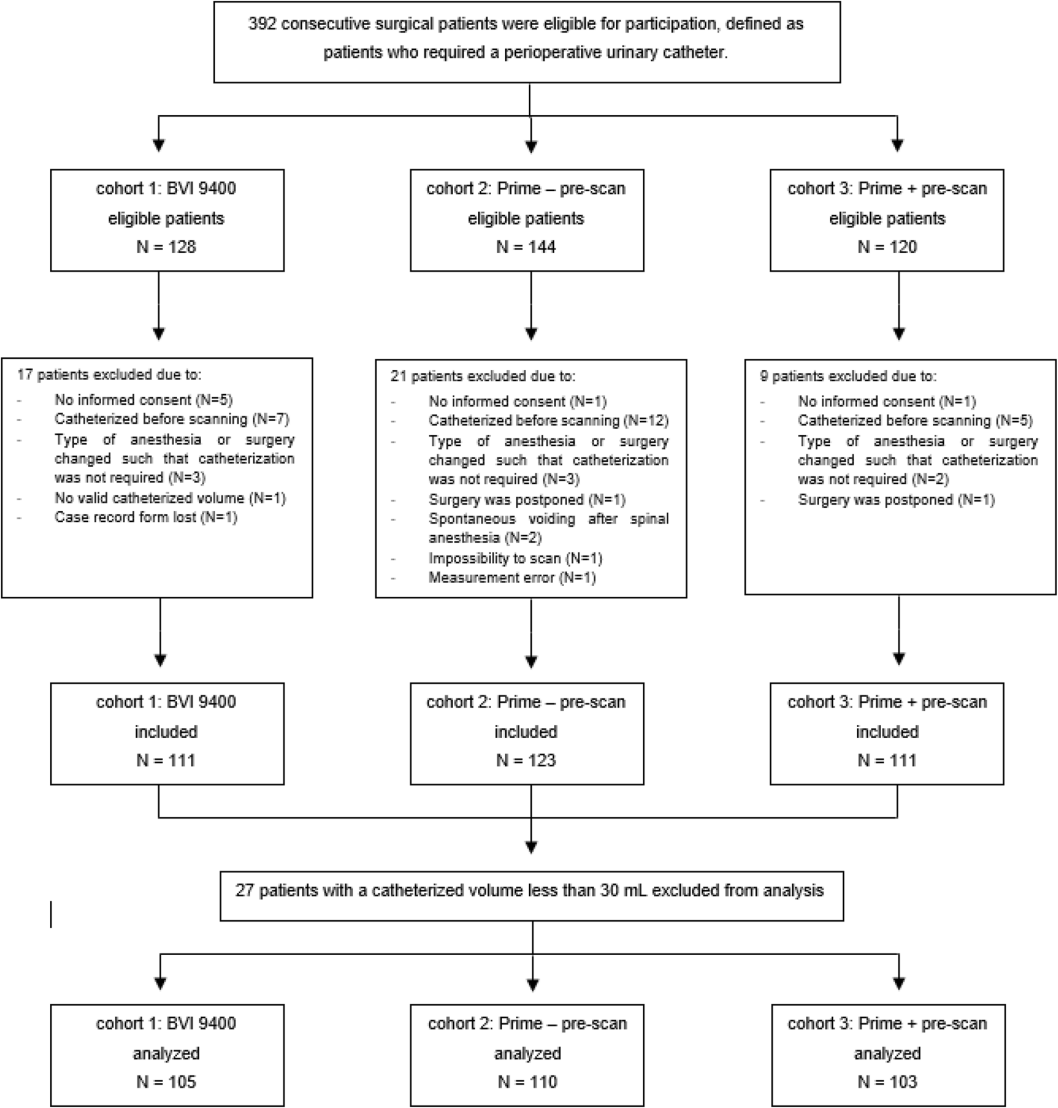
Flow-chart patient selection

Between June 2016 and September 2016, 392 consecutive patients were evaluated for participation in this study of which 345 were included. Of these, 318 patients were available for analysis (Fig. 1, Flow Chart).

Table 1 presents the demographic characteristics of the patients included. No statistically significant differences between the cohorts were observed at baseline, except for number of patients measured postoperatively. In the cohort of the BVI 9400® significant more patients were assessed postoperatively compared to the cohort of both Primes (15, 3 and 9% respectively, p = 0.003). In addition, measured bladder volumes (estimated and actual) are presented for all three cohorts.

**Table 1:** Demographic characteristics

Patient data	BVI 9400 (n = 105)		Prime *without* pre-scan (n = 110)		Prime *with* pre-scan (n = 103)		p value
Women no. (%)	52 (49.5)		42 (38.2)		47 (45.6)		0.23
Age mean (SD) (year)	65.9 (13.4)		66.6 (12.5)		64.5 (12.4)		0.41
Age < 60, no. (%)	31 (29.5)		26 (23.6)		29 (28.2)		0.61
Weight (mean) (kg)	80.4 (17.0)		82.9 (15.5)		82.0 (14.7)		0.37
Height (mean) (cm)	172.4 (9.3)		174.3 (9.1)		174.5 (8.9)		0.14
BMI (mean) (kg/m^2^)	27.0 (4.8)		27.2 (4.2)		27.0 (4.6)		0.88
BMI < 25 no. (%)	38 (36.2)		40 (36.4)		38 (36.9)		0.97
≥25 BMI < 30 no. (%)	44 (41.9)		45 (40.9)		39 (37.9)		
BMI ≥ 30 no. (%)	23 (21.9)		25 (22.7)		26 (25.2)		
Uterus no. (%)	46 (88.5)		37 (88.1)		39 (83.0)		0.71

*BMI*  body mass index

### Primary outcome

#### Equivalence of bladder volume estimates

For the primary endpoint of equivalence of bladder volume estimates, in all three cohorts the equivalence was not established. Over the whole volume range, the BVI 9400® *over*estimated the actual bladder volume by an average of + 17.5% (95% CI + 8.77 to + 26.31, p = 1.00). The Prime® *without* pre-scan underestimated the actual bladder volume by an average of − 4.1% (95%CI − 8.78 to + 0.49, p = 0.36) and the Prime® *with* pre-scan *under*estimated the actual bladder volume by an average of − 6.3% (95% CI − 11.57 to − 1.07, p = 0.69) (Table 2).

**Table 2:** Actual and estimated bladder volumes measured

	BVI 9400 (n = 105)	Prime *without* pre-scan (n = 110)	Prime *with* Pre-scan (n = 103)	p value
Estimated volume, mean (SD) (mL)	288 (237.0)	191 (163.7)	203 (182.1)	< 0.001
Actual volume, mean (SD) (mL)	266 (241.9)	212 (202.8)	224 (226.6)	0.18
Difference estimated–actual volume, mean (SD) (mL)	21.8 (59.9)	− 20.7 (70.1)	− 20.7 (82.7)	< 0.001
Preoperative measurement, no (%)	89 (84.8)	107 (97.30)	94 (91.3)	0.005
Actual volumes > 400 mL, no (%)	26 (24.8)	15 (13.6)	16 (15.5)	0.078
Actual volumes > 500 mL, no (%)	17 (16.2)	9 (8.2)	14 (13.6)	0.194
Actual volumes > 600 mL, no (%)	11 (10.5)	4 (3.6)	8 (7.8)	0.149

#### Level of agreement

**Fig. 2:**
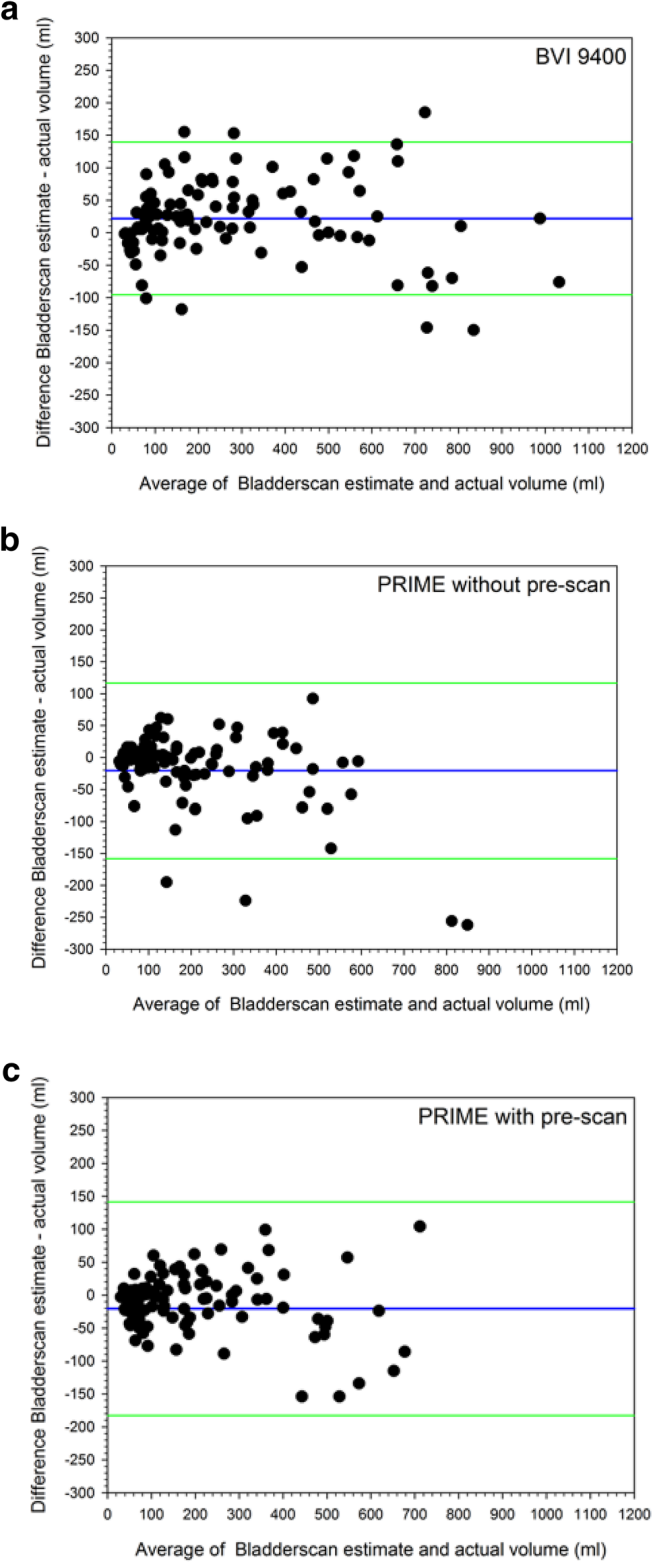
**a** Bland–altman plot displaying the level of agreement of the BladderScan® Bvi 9400 over the whole volume range. **b** Bland–altman plot displaying the level of agreement of the prime® *without pre-scan* over the whole volume range. **c** Bland–altman plot displaying the level of agreement of the prime® *with pre-scan* over the whole volume range. Plotted is the average of the measured volume plus the scanned volume (X-axis), versus the difference of the measured volume minus the scanned volume (Y-axis). Blue horizontal line indicates the mean difference (= bias), and the green horizontal lines indicate two times standard deviation (= precision)

The BVI 9400® *over*estimates the actual bladder volume with a bias of + 21.8 mL and limits of agreements (LOA) of − 99 mL to + 140 mL Both Prime® devices demonstrated a significant bias towards *under*estimation (both *with* and *without* performing a pre-scan, bias = − 20.7 mL) and wide limits of agreement (− 183 to + 141 mL) (Fig. 2).

### Secondary outcomes

#### Subgroup analyses for homogeneity of BladderScan® performance

As presented in Table 3, subgroup analysis for the homogeneity of the performance of the BladderScan® over different subgroups, showed that equivalence was not reached in any of the predefined subgroups. Although not reaching equivalence, some differences between subgroups were observed. For example, for the Prime® *without* and *with* pre-scan function, the *under*estimation in male patients was − 0.8 and + 0.2% respectively, almost reaching equivalence (p = 0.08). In patients with a BMI < 25 kg/m^2^ both Primes modes performed well, with an *under*estimation of − 0.5 and − 2.7% respectively, but still not reaching equivalence (p = 0.112 and 0.266).

**Table 3:** primary and secondary outcomes and differences in percentages by patient characteristics and bladder volume ≤ 400 and > 400 ml

	BVI 9400			Prime *without* pre-scan			Prime *with* pre-scan		
	Mean	95% CI	p	Mean	95% CI	p	Mean	95% CI	p
Overall	17.5 (45.3)	8.77 to 26.31	0.997	− 4.1 (24.5)	− 8.78 to 0.49	0.357	− 6.3 (26.9)	− 11.57 to − 1.07	0.690
Male	19.8 (49.5)	6.18 to 33.48	0.983	− 0.8 (24.5)	− 6.71 to 5.16	0.080	0.2 (25.6)	− 6.67 to 7.03	0.082
Female	15.2 (41.0)	3.80 to 26.61	0.961	− 9.6 (23.8)	− 17.02 to − 2.17	0.891	− 14.1 (26.5)	− 21.85 to − 6.261	0.998
Age > 60 year	16.4 (33.9)	3.95 to 28.82	0.964	− 2.1 (26.6)	− 12.84 to 8.61	0.292	− 7.2 (25.7)	− 16.93 to 2.61	0.673
Age ≤ 60 year	18.0 (49.5)	6.55 to 29.50	0.987	− 4.8 (24.0)	− 9.98 to 0.44	0.465	− 6.0 (27.5)	− 12.35 to 0.38	0.621
BMI ≤ 25	20.2 (56.1)	1.77 to 38.66	0.948	− 0.5 (22.8)	− 7.83 to 6.74	0.112	− 2.7 (22.7)	− 10.13 to 4.77	0.266
25 < BMI ≤ 30	14.4 (45.6)	0.54 to 28.24	0.911	− 6.4 (25.2)	− 13.99 to 1.12	0.648	− 8.3 (28.7)	− 17.58 to 1.05	0.759
BMI > 30	19.1 (17.9)	11.39 to 26.90	0.999	− 5.8 (26.4)	− 16.68 to 5.12	0.558	− 8.7 (30.0)	− 20.82 to 3.40	0.733
≤ 400 mL	22.2 (50.9)	10.75 to 33.55	0.998	− 2.7 (25.1)	− 7.86 to 2.38	0.191	− 5.0 (28.3)	− 11.0 to 1.05	0.497
> 400 mL	3.5 (13.8)	− 2.04 to 9.10	0.296	− 13.1 (18.6)	− 23.32 to − 2.78	0.943	− 13.6 (16.0)	− 22.16 to − 5.08	0.976

Numbers in percentages (%) + standard deviation (SD)

Age in years

*BMI * body mass index (kg/m^2^), *CI* confidence interval

For the BVI 9400® used in patients with smaller or larger bladder volumes than 400 mL the *over*estimation decreased from + 22% in patients with an actual bladder volume ≤ 400 mL (N = 79) to + 3.5% in patients with an actual bladder volume > 400 mL (N = 26). In contrast, in the Prime® *without* pre-scan the *under*estimation increased from − 2.7% in patients with an actual bladder volume ≤ 400 mL (N = 95) to − 13.1% in patients with an actual bladder volume > 400 mL(N = 15). For the Prime® *with* pre-scan the *under*estimation increased from − 5.0% in patients with an actual bladder volume ≤ 400 mL (N = 87) to − 13.6% in patients with an actual bladder volume > 400 mL (N = 16).

Addressing the level of agreement using the Bland–Altman method showed the same results: the BVI 9400® showed less bias with bladder volumes > 400 mL (bias = + 10.0 mL) than with smaller bladder volumes ≤ 400 mL (bias = + 25.7 mL). For the Prime® device in both modes this was reversed: in patients with bladder volumes ≤ 400 mL the bias was − 7.9 and − 4.0 mL and for patients with bladder volumes > 400 mL the bias was − 101.5 and − 111.8 mL for the Prime® *without* and *with* pre-scan respectively (Table 4).

**Table 4:** Bland–altman analysis over the two volume ranges ≤ 400 and > 400 mL by type of BladderScan®

	Volumes ≤ 400 mL		Volumes > 400 mL			
	N	Bias	LOA	N	Bias	LOA
BVI 9400	79	25.7	− 69 to 121	26	10.0	− 159 to 179
Prime *without* pre-scan	95	− 7.9	− 81 to 65	15	− 101.5	− 385 to 182
Prime *with* pre-scan	87	− 4.0	− 72 to 64	16	− 111.8	− 447 to 223

Bias = average of actual volume ^ scanned volume (mL)

*LOA* level of agreement (mL)

#### Consequences for clinical decision-making

With the BVI 9400®, the proportion true negative (= bladder volume measured and actual bladder volume both ≤ 500 mL) and true positive (= bladder volume measured and actual bladder volume both > 500 mL) clinical decisions whether or not to catheterize the bladder (using a threshold of 500 mL) was 1.00 (95%CI 0.96–1.00) and 0.91 (95% CI 0.79–1.00), respectively. Comparable results were found with the Prime® *without* pre-scan 0.96 (95% CI 0.92–1.00) and 0.86 (95% CI 0.60–1.00) respectively and the Prime® *with* pre-scan 0.94 (95%C I 0.87–0.98) and 1.00 (95% CI 0.64–1.00) respectively (Table 5).

**Table 5:** The proportion well predicted clinical decisions whether or not to catheterize at a threshold of 400, 500, and 600 ml, by type of BladderScan®

	400 mL		500 mL		600 mL	
	True negatives	True positives	True negatives	True positives	True negatives	True positives
BVI 9400	100 (1.00–1.00)	89.7 (0.79–1.00)	100 (1.00–1.00)	90.9 (0.79–1.00)	98.9 (0.97–1.00)	80.0 (0.60–1.00)
Prime *without* pre-scan	97.9 (0.95–1.00)	87.5 (0.71–1.00)	96.1 (0.92–1.00)	85.7 (0.60–1.00)	98.1 (0.96–1.00)	100 (1.00–1.00)
Prime *with* pre-scan	97.7 (0.94–1.00)	82.4 (0.64–1.00)	93.7 (0.87–0.98)	100 (1.00–1.00)	96.0 (0.92–1.00)	100 (1.00–1.00)

Data are presented as percentage (95% confidence interval)

## Discussion

In this study, the performance of the BladderScan BVI 9400® and its successor Prime® were assessed in surgical patients, both perioperative and postoperative. The estimates of bladder volumes were compared with actual bladder volumes collected after catheterization, which is considered the “gold standard” for measuring urinary volumes.

We a-priori set the margin of equivalence for a clinically acceptable estimation of the bladder volume to ± 5%, based on the literature in which a mean difference of − 7% with the BVI 2500® and a mean difference of − 3.3% with the BVI 3000®, an earlier version of the BladderScan, were observed [13, 15]. In neither the BVI 9400® nor the Prime® (*with* or *without* pre-scan option used) the performance was within our margin of equivalence. In this, the BVI 9400® structurally overestimated the actual bladder volume and the Prime® *without* and *with* pre-scan structurally *under*estimated actual bladder volume. *Under*estimation may be preferred considering the fact that postoperative catheterization is a complication patients would like to avoid, but only if a strict “bladder protocol” is used to prevent bladder overdistension. *Over*estimation of the actual bladder volume will lead to earlier and more unnecessary postoperative urinary catheterizations [20].

When using the manufacturer’s claim of an accuracy (± 15% ±15 mL) for the BVI 9400® and the Prime®, the amount of *over*estimation of the BVI 9400® was still beyond that level of accuracy. For the Prime® (both *with* or *without* pre-scan) the amount of *under*estimation was within the manufacturers’ claim.

In clinical practice, a measured bladder volume of 500 mL, which is considered a clinical cut-off value for bladder catheterization, should ideally not deviate more than 5% from the true bladder volume (i.e., between 475 and 525 mL), because in this range the BladderScan® can help the clinician in clinical decision-making. The manufacturer claims an accuracy of ± 15% ± 15 mL, both for the BVI 9400® and the Prime® (i.e. between 410 and 590 mL). This is a rather large volume range, especially when the decision is whether or not to catheterize.

A possible explanation of these differences in the results between the BVI 9400® (overestimating and performing better in bladder volumes > 400 mL), and both Prime’s (underestimating and performing better in bladder volumes ≤ 400 mL) could be that the BVI 9400® is using another, older algorithm, based on earlier devices such as the BVI 3000®. The Prime® uses a new developed algorithm, which has only been tested in phantoms in the factory and not on patients.

When expressing the error in BladderScan® estimates in millilitres, using the Bland–Altman method, the mean bias was relatively small (+ 22 mL for the BVI 9400® and − 21 mL for both modes of the Prime®). Previous studies investigating the accuracy of older models of the BVI (BVI 2500®/2500®+, BVI 3000®) have reported similar mean errors (bias) ranging from − 21.5 to + 19 mL [13–15]. However, the limits of agreement in both the BVI 9400® and Prime® were large in bladder volumes > 400 mL (range > 340 mL for all three scanners) indicating a high variability between individual subjects towards both *under*- and *over*estimation. This was also reflected in the lack of equivalence in all three cohorts.

Additional subgroup analyses did not show significant differences between volumes ≤ 400 and > 400 mL. However, in patients with bladder volumes > 400 mL the BVI 9400® tends to perform better with an average *over*estimation of 3.5% compared to 17.5% over the entire volume range. In the Prime® this was not observed; the average *under*estimation increased when measuring bladder volumes > 400 mL (to almost − 14%). Although not reaching statistical significance, these results suggest that BVI 9400® estimates are more accurate in the higher volume range, whereas the Prime® appears to perform better in the lower volume range. Previous studies also reported higher discrepancies between the BladderScan® estimate and catheterization in actual bladder volumes of less than 50–100 mL and larger than 400mL^19^. Nonetheless, in all three BladderScans the large variability did not have a strong impact on the occurrence of unnecessary (true positives ≥ 80%) or missed (true negatives ≥ 94%) catheterizations, although the numbers of patients with higher bladder volumes were too small to draw meaningful conclusions.

We expected that the Prime® *with* pre-scan ability would improve the accuracy of the measured bladder volume. Using real-time echo image should help to scan the whole bladder. In clinical practice this image was unsteady, and it was difficult to encircle the whole bladder with the green line. Therefore, the pre-scan function felt unreliable. It was surprising to find minimal differences between the Prime® *without* and *with* pre-scan. In fact, slightly better results were observed when using the Prime® *without* pre-scan, suggesting that using the pre-scan feature does not improve accuracy.

We also demonstrated that age or BMI did not significantly affect the accuracy of the BladderScan®. However, with the Prime®, we observed more accurate results in males. Previous studies also found that the BladderScan® (BVI 2500®/2500®+) was more accurate in males than females [13, 21], suggesting that the algorithm for female patients may need to be improved. Also, in patients with small BMI’s (< 25 kg/m^2^) the BVI 9400® had difficulty in scanning the entire bladder (mean + 20.2 mL) particularly with larger bladder volumes. The display of the device and its printouts showed that parts of the bladder were outside the scanning area with measured values labeled “larger than” (>) instead of “is” (=). Possibly, in lean patients the scanhead is positioned too close to the bladder, which prevents the device from scanning the whole bladder. This was not the case with the Prime®. In female patients and patients with a BMI ≥ 25 kg/m^2^ these effects were less pronounced. Overall, our results suggest that the newer algorithms are more accurate in subgroups of patients (male patients and patients with a BMI < 25 kg/m^2^), but still need improvement.

The strength of this study is that it was performed in a large number of unselected patients requiring bladder catheterization in a daily clinical practice setting. The same trained researcher (CvdB) performed more than 80% of the measurements eliminating inter-observer bias. This might potentially limit the generalizability of our findings for settings where the measurements are performed by many different nurse operators—with varying experience in bladder ultrasound scanning. However, inter-observer reliability has been investigated in several previous studies and was found to be high [22–24]. Previous studies also showed that repeated measures do not improve the accuracy of the BladderScan® and that the results are independent of patient position and experience of the examiner [24, 25]. Another limitation of our study is that we did not include many patients with bladder volumes larger than 500 mL. Therefore, our findings with regard to the performance of the BladderScan® in the higher volume ranges and in correct clinical decision-making, need to be interpreted with caution.

## Conclusion

This study showed that the ultrasound devices BVI 9400® and Prime® *with* and *without* pre-scan were not able to measure the actual bladder volume within our strict predefined limits of ± 5%. However, the BVI 9400® performed better in bladder volumes > 400 mL and the Prime® performed better in bladder volumes ≤ 400 mL, in male patients and in patients with a BMI < 25 kg/m^2^. The pre-scan feature of the Prime® did not improve accuracy. Taken together, this suggests that the algorithms for measuring bladder volume non-invasively can be further improved, helping the clinician in making the right decision whether or not to catheterize, not too early (= unnecessary) but certainly not too late (= preventing bladder distention). An important lesson learnt is that new devices should be validated in clinical practice, on real patients, before they are sold to the market. On the other hand, despite their limited accuracy, the studied devices adequately facilitated clinical decision making when the decision is at hand whether it is necessary to catheterize the patient, especially when bladder volumes were 500 mL or more.
